# Full-Thickness Skin Transplantation Combined with Negative-Pressure Wound Therapy for a Refractory Peristomal Large Skin Ulcer Caused by Mucocutaneous Separation: A Case Report

**DOI:** 10.70352/scrj.cr.25-0725

**Published:** 2026-04-01

**Authors:** Atsushi Okita, Toshiyuki Watanabe, Mari Nakagawa, Kaori Ogino, Shinsuke Hashida, Kazunori Tsukuda, Nobuji Yokoyama

**Affiliations:** 1 Department of Surgery, Okayama City Hospital, Okayama, Okayama, Japan; 2 Department of Surgery, Setouchi City Hospital, Setouchi, Okayama, Japan; 3 Department of Plastic Surgery, Okayama City Hospital, Okayama, Okayama, Japan; 4 Department of Surgery, Shinmatsudo Central General Hospital, Matsudo, Chiba, Japan; 5 Department of Nursing, Senoo Hospital, Okayama, Okayama, Japan

**Keywords:** peristomal skin ulcer, mucocutaneous separation, skin transplantation, negative-wound pressure therapy

## Abstract

**INTRODUCTION:**

Stomal mucocutaneous separation is a frequent complication of colostomy, which may complicate wound care due to contamination from fecal leakage, resulting in delayed wound healing. In addition, systemic factors may further influence postoperative wound healing. We report successful combination treatment, involving full-thickness skin graft transplantation and negative-pressure wound therapy (NPWT) in a patient with a large peristomal ulcer that developed progressively in the postoperative course following complete circumferential mucocutaneous separation and infection, which occurred after neoadjuvant chemotherapy followed by laparoscopic abdominoperineal resection.

**CASE PRESENTATION:**

A 75-year-old male, who had been diagnosed with advanced low rectal cancer, received 4 cycles of mFOLFOX6 chemotherapy (oxaliplatin, leucovorin, and fluorouracil) plus bevacizumab, followed by laparoscopic abdominoperineal resection with lymph node dissection. Approximately 4 weeks later, partial mucocutaneous separation developed, accompanied by subcutaneous abscess formation. Subsequently, this led to complete circumferential mucocutaneous separation, which evolved into a giant peristomal ulceration with a significant tissue loss. Conservative local wound care, including frequent debridement, was continued, and granulation tissue gradually filled the peristomal skin defect. Three months after the operation, full-thickness skin graft transplantation combined with NPWT was performed. At 6 years after the operation, the patient remained recurrence-free, and there were no postoperative complications or issues with the colostomy or skin graft.

**CONCLUSIONS:**

This combination therapy successfully treated a giant peristomal ulcer that progressively developed after mucocutaneous separation with infection, which occurred later than typical postoperative wound complications following neoadjuvant chemotherapy (mFOLFOX6 + bevacizumab) and laparoscopic surgery. Delayed wound healing, potentially related to bevacizumab, may have contributed to both the late onset and unusually extensive progression of the ulcer. The therapy proved compatible with long-term stoma care.

## Abbreviations

NPWT
: negative-pressure wound therapy
SSI
: surgical site infection
VEGF
: vascular endothelial growth factor

## INTRODUCTION

Stoma creation is a common procedure in colorectal surgery. However, the frequency of ostomy complications is high, with nearly 80% of ostomy patients experiencing complications in the early postoperative period.^1)^ Early complications, which occur within the first 30 days after the stoma creation, include ischemia/necrosis, retraction, mucocutaneous separation, and parastomal abscess.^2)^ Among them, stomal mucocutaneous separation is a frequent complication, occurring in 12% to 24% of patients in the early postoperative period.^3)^ Once mucocutaneous separation occurs, wound care becomes complicated due to contamination from fecal leakage, and wound healing takes longer. Herein, we report successful combination treatment with full-thickness skin graft transplantation and NPWT in a patient diagnosed with low rectal cancer who progressively developed a large peristomal ulcer with delayed wound healing after complete circumferential mucocutaneous separation and infection, which arose after laparoscopic abdominoperineal resection and neoadjuvant chemotherapy with mFOLFOX6 (oxaliplatin, leucovorin, and fluorouracil) plus bevacizumab.

## CASE PRESENTATION

A 75-year-old male, with a history of hyperuricemia visited our outpatient clinic following a positive fecal occult blood test during colorectal cancer screening. The patient was diagnosed with low rectal cancer, which was staged as cT4aN1M0 (stage IIIb), according to the 8th edition of the Union for International Cancer Control TNM classification. He underwent totally implantable central venous access port placement and received 4 cycles of mFOLFOX6 plus bevacizumab. Four weeks after the completion of the chemotherapy, he underwent laparoscopic abdominoperineal resection with lymph node dissection.

The patient’s preoperative clinical and laboratory findings were as follows: he had a BMI of 23.0 kg/m^2^. In addition, his laboratory data revealed a white blood cell count of 4740/μL (normal range: 3300–8600/μL), a red blood cell count of 4.46 × 10^6^/μL (normal range: 4.35–5.55 × 10^6^/μL), a hemoglobin level of 13.0 g/dL (normal range: 13.7–16.8 g/dL), a platelet count of 32.4 × 10^4^/μL (normal range: 15.8–34.8 × 10^4^/μL), a total bilirubin level of 0.36 mg/dL (normal range: 0.4–1.5 mg/dL), an aspartate aminotransferase level of 19 IU/L (normal range: 13–30 IU/L), an alanine aminotransferase level of 23 IU/L (normal range: 10–42 IU/L), an albumin level of 3.8 g/dL (normal range: 4.1–5.1 g/dL), a blood urea nitrogen level of 9 mg/dL (normal range: 8–20 mg/dL), a creatinine level of 0.80 mg/dL (normal range: 0.65–1.07 mg/dL), a C-reactive protein level of 0.35 mg/dL (normal range: 0–0.14 mg/dL), and a hemoglobin A1c level of 5.8% (normal range: 4.9%–6.0%).

**Fig. 1:**
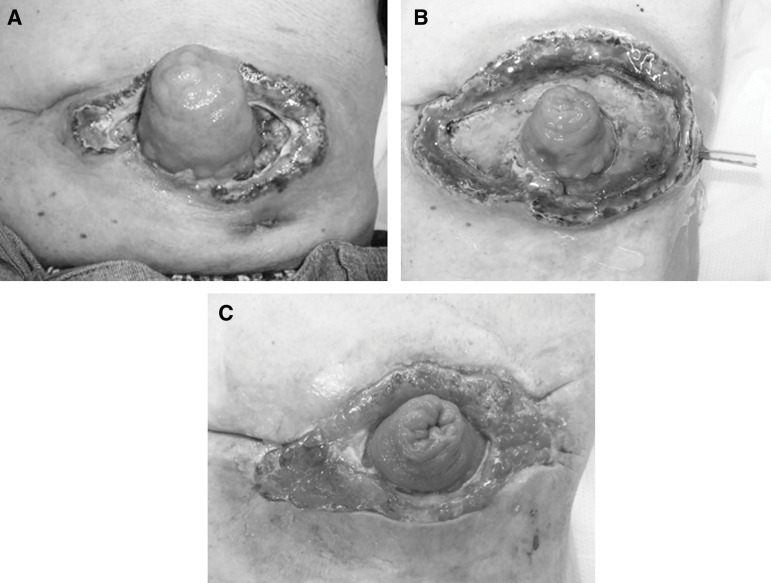
(**A**) The appearance of the stoma on readmission. (**B**) The appearance of the stoma before the 4th wound debridement. (**C**) The appearance of the stoma before the operation.

Three weeks after the operation, the peristomal skin became irritated and red. Partial mucocutaneous separation developed on the 26th POD, and peristomal skin necrosis, accompanied by subcutaneous fat necrosis and purulent discharge, gradually spread around the stoma. Despite no improvement in the condition of the peristomal skin, the patient was discharged home, as per his strongly expressed wishes, on the 36th POD. On the 44th POD, he was rehospitalized due to an inability to manage his colostomy, resulting from extensive mucocutaneous separation with peristomal skin necrosis and the formation of a subcutaneous pocket. The peristomal ulcer measured 12 × 5 cm. Upon readmission, nearly the entire circumference of the stoma showed mucocutaneous separation with skin necrosis (**Fig. 1A**). Total parenteral nutrition with fasting was introduced to reduce the amount of stomal output. The skin defects were managed with cadexomer iodine paste (Cadex Ointment 0.9%; Smith & Nephew, Hertfordshire, UK), gauze dressing soaked in saline, AQUACEL Ag+ dressings (Convatec, Greensboro, NC, USA), and Mepilex Ag (Mölnlycke Health Care, Mölndal, Sweden), onto which a stoma flange was applied. Wound care was collaboratively managed with the primary nurses, with support from a nurse certified in Wound, Ostomy, and Continence Nursing by the Japanese Nursing Association. In addition, debridement with excision of the peristomal necrotic skin was performed 4 times between the 47th and 62nd POD (**Fig. 1B**). Granulation tissue gradually filled the peristomal skin defect, but the ulcer was large (**Fig. 1C**). Bacterial cultures of the peristomal ulcer obtained on the 79th POD identified *Klebsiella oxytoca*, *Citrobacter freundii*, *Enterococcus faecalis*, and methicillin-resistant *Staphylococcus aureus*. However, there were no clinical signs of an active infection. Because stoma care was difficult for the patient to manage independently, leading to long-term impairment of his ability to perform ADL, it was determined that a skin graft was necessary. Full-thickness skin grafting combined with NPWT was performed on the 90th POD.

### Surgical procedures and NPWT

**Fig. 2:**
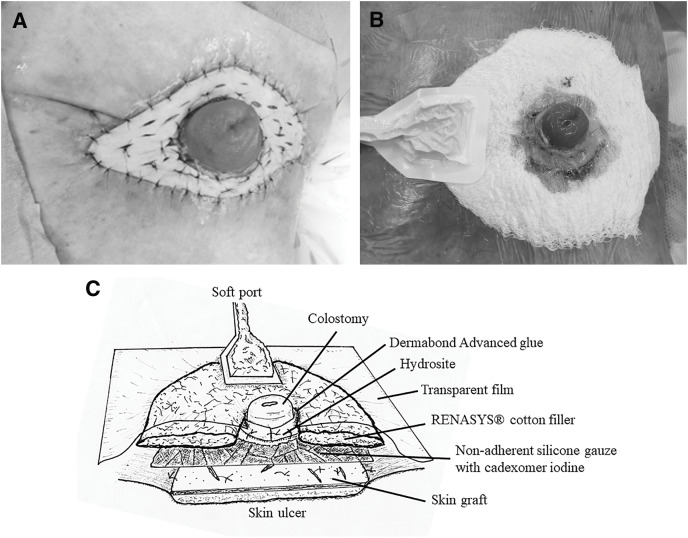
(**A**) The skin graft was fixed on the peristomal skin ulcer. (**B**) NPWT. (**C**) Schematic illustration of the NPWT dressing placed over the peristomal ulcer covered with a skin graft. NPWT, negative-pressure wound therapy

Under general anesthesia, the patient was placed in the supine position. The skin defect measured 13 × 8 cm. A full-thickness skin graft was harvested from the left thigh. Excess defective granulation tissue was scraped away from the peristomal skin ulcer, and the wound was flattened. The skin graft taken from the left thigh was placed over and fixed onto the peristomal skin ulcer using 5-0 Monocryl sutures (Ethicon, Somerville, NJ, USA) (**Fig. 2A**). A polyurethane foam dressing (Hydrosite thin type; Smith & Nephew) was cut into strips, wrapped around the base of the colostomy, and sutured to secure it in place. Non-adherent silicone-treated gauze (TREX; Fuji Systems, Tokyo, Japan) was cut into small pieces, and cadexomer iodine paste (Smith & Nephew) was applied to it. These were then placed on the transplanted skin graft. The RENASYS GO NPWT system (Smith & Nephew) was used for the NPWT. RENASYS cotton filler was cut to cover the skin graft, and a center hole was made for the colostomy. The cotton filler was placed accordingly. A transparent film with a center hole for the colostomy was placed over the cotton filler. The edges of the hole in the film were sutured to the Hydrosite at the base of the colostomy. Dermabond Advanced (Ethicon) glue was applied to seal the gaps between the edges of the hole in the film and the Hydrosite at the base of the colostomy. A hole was cut in the film, and a soft port was placed over it (**Fig. 2B**). The tubing of the soft port was connected to the canister tubing of the NPWT system. The NPWT system was set to continuous mode at a negative pressure of 100 mmHg. A schematic illustration of the NPWT dressing configuration around the stoma is provided in **Fig. 2C**. The operation time was 1 h and 58 min, and the amount of intraoperative blood loss was 70 g.

### Postoperative course

**Fig. 3:**
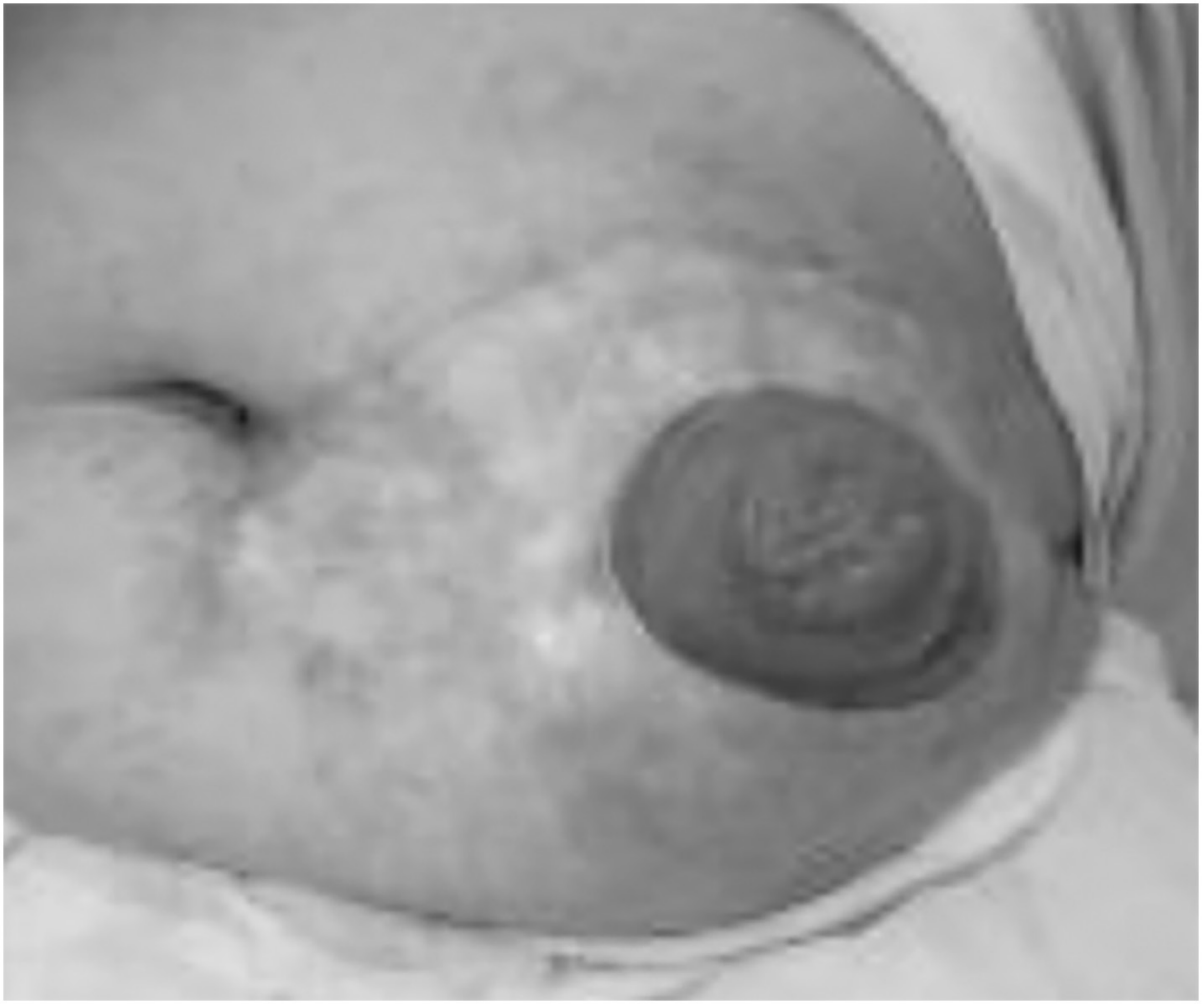
The appearance of the stoma 6 years after the operation.

The patient’s postoperative clinical course was uneventful, and the NPWT was completed on the 7th POD. Minor wound care and stoma care were performed. The patient was discharged on the 28th POD. There was no postoperative recurrence or issues with the colostomy or skin graft at 6 years after the operation (**Fig. 3**).

## DISCUSSION

We reported a rare case in which a patient with low rectal cancer developed a large peristomal ulcer due to complete circumferential mucocutaneous separation and infection after neoadjuvant chemotherapy with mFOLFOX6 plus bevacizumab and laparoscopic abdominoperineal resection, and was treated with full-thickness skin graft transplantation and NPWT.

Mucocutaneous separation is defined as the partial or circumferential detachment of the mucosa from the peristomal skin with or without an associated abscess.^4)^ The depth of such separation can vary and is classified as superficial if it only involves the epidermis or deep if it involves the dermis and subcutaneous layers.^4)^ When mucocutaneous separation is accompanied by infection, it can be regarded as a type of superficial SSI. The occurrence of SSIs is influenced by multiple factors, including patient- and treatment-related variables.^5)^ In our case, patient-related factors, such as comorbid diseases, did not appear to affect the SSI, but treatment-related factors, including colostomy creation,^6)^ were considered to be involved. Although recent studies have not reported an association between neoadjuvant chemotherapy and SSI,^7,8)^ neoadjuvant chemotherapy has been identified as a risk factor for mucocutaneous separation.^9)^

Our patient received neoadjuvant chemotherapy including bevacizumab. Bevacizumab is approved and is widely used in combination with chemotherapy for unresectable metastatic colorectal cancer.^10)^ In contrast, its use in the neoadjuvant setting for locally advanced rectal cancer, with the aim of achieving local and distant tumor control, has been investigated in several clinical studies.^11)^ Although evidence regarding neoadjuvant chemotherapy with bevacizumab remains limited and this approach cannot be regarded as standard therapy, previous studies have suggested that the addition of bevacizumab may improve tumor responses and local disease control in selected patients with locally advanced rectal cancer.^11)^ Based on these considerations, this treatment strategy was adopted in the present case.

Bevacizumab-containing chemotherapy administered prior to surgery may negatively impact surgical wound healing.^12)^ VEGF plays a key role in wound healing through 3 major mechanisms: vasodilation, increased vascular permeability, and angiogenesis.^13)^ Bevacizumab is a humanized monoclonal antibody that selectively binds to VEGF-A, thereby inhibiting these processes, and may lead to wound-healing complications.^12,13)^ A previous study demonstrated that anti-VEGF therapy is associated with impaired wound healing in the skin and gastrointestinal tract, including bowel dehiscence and wound-healing complications.^12)^ In that study, the overall rate of wound-healing complications after preoperatively administered bevacizumab (within 0–60 days before surgery) was 13%, and the rate of such complications among patients who underwent surgery between 31 and 60 days after the administration of bevacizumab was 6.7%.^12)^ In our case, the interval between the last bevacizumab dose being administered and the operation was 4 weeks. Although the patient’s other surgical wounds had healed, the peristomal lesion developed on the 26th POD, which is relatively late for a typical SSI, and subsequently progressed, resulting in extensive skin necrosis that could not be fully explained by an infection alone. The patient’s preoperative clinical and laboratory findings, including BMI, blood counts, the albumin level, and the hemoglobin A1c level, were within normal or near-normal ranges, suggesting that patient-related factors were unlikely to have significantly contributed to the peristomal lesion. While an infection may have played a role, the potential influence of bevacizumab on delayed peristomal wound healing cannot be excluded, as the surgery was performed 4 weeks after the last dose was administered, and the abovementioned study reported wound-healing complications occurring when surgery was performed within 60 days of bevacizumab administration.^12)^ Therefore, the peristomal lesion may have been a drug-related skin disorder associated with bevacizumab.

Conservative therapy for mucocutaneous separation typically involves local wound care, such as irrigation with saline, the use of skin-barrier powder to absorb any exudate and fill the defect, and more frequent replacement of the stoma device.^4,14)^ In cases of deep mucocutaneous separation or superficial mucocutaneous separation in which conservative treatment fails, surgical treatment is required.^4)^ In our case, the re-creation of a colostomy through a reoperation would have posed similar risks, and the skin defect surrounding the colostomy was extensive; therefore, conservative treatment was initially chosen. A similar case was reported by Taira et al., in which a large skin ulceration and delayed wound healing around a colostomy occurred in a patient with metastatic rectal cancer who was receiving VEGF-targeting therapy.^15)^ This case was successfully treated with conservative management, and the associated report was the first to detail skin toxicity around a colostomy following VEGF-targeting therapy.^15)^ In contrast, in our case, the ulcer continued to expand due to surrounding skin necrosis, and conservative therapy alone was insufficient for healing. Moreover, the healing of the peristomal ulcer had already been prolonged because time was required for the surrounding necrotic tissue to demarcate and stabilize without further progression. As the patient’s QOL had been significantly impaired by the peristomal ulcer, continued conservative therapy alone was considered unlikely to achieve wound healing, and the course of such healing was likely to be unpredictable.

NPWT was developed in the 1990s and has been used to treat problematic wounds.^16–18)^ NPWT is employed for various types of acute and chronic wounds, including pressure wounds, diabetic leg ulcers, lower leg wounds, surgical incisions, traumatic wounds, burns, infected wounds, necrotizing fasciitis, infected sternal wounds, and post-skin grafting wounds.^18)^ Previous systematic reviews have suggested that NPWT may have a positive effect on wound healing compared with conservative therapy; however, due to heterogeneity or bias among studies, the superiority of NPWT for achieving complete wound closure remains unclear.^19,20)^ Therefore, NPWT alone may not reliably guarantee complete wound healing. NPWT also has a beneficial perioperative role for skin grafting, as it prepares the optimal wound bed for skin graft acceptance and stabilizes the graft by reducing exudate to lower the risk of hematoma and seroma formation, while increasing granulation to facilitate revascularization and attachment of the graft, thereby enhancing post-graft adherence and survival.^21)^ In the present case, we considered that, while NPWT alone cannot reliably achieve complete wound healing, it was effective as an adjunct to support skin graft procedures. Skin grafting is one of the most common surgical procedures for non-healing wounds and may be considered a suitable solution in such cases, especially when wounds fail to heal after approximately 6 weeks.^22)^ However, in our case, the wound environment was unfavorable due to the presence of a colostomy. Therefore, NPWT was used to create a favorable environment for successful skin grafting.

The use of NPWT as an adjunct to skin grafting has been increasingly recognized in clinical practice. The clinical use of NPWT after skin grafting was first introduced in1990.^23)^ While NPWT has conventionally been used for treating open wounds, it is increasingly applied to closed wounds, fluid management, and as an adjunct to improve skin graft adherence for both meshed and sheet grafts.^24)^ NPWT has been shown to reduce graft failure rates, hospital stays, reoperation rates, and complications, while also improving patient satisfaction.^24)^ There are only 3 reports on the use of NPWT for peristomal wounds,^25–27)^ and 2 of them, which were published in 2010 and 2014, respectively, described the combined use of NPWT with skin graft transplantation.^25,26)^ Clavijo-Alvarez reported the case of an 82-year-old woman who developed a peristomal wound caused by folliculitis about 10 years after the creation of a colostomy for diverticulitis.^25)^ The ulcer progressed over 2 months to a 12 × 6-cm horseshoe-shaped area around the stoma. Once the wound condition had stabilized, meshed skin grafting combined with NPWT was performed with the goal of achieving rapid epithelialization of the surrounding area prone to stool contamination. When the dressing was removed 4 days later, 98% of the graft had taken, resulting in successful wound healing. Byrnes et al. reported a series of 7 patients with wounds adjacent to enterocutaneous fistulas, including 4 females and 3 males, with a mean age of 60.8 years (range: 34–79).^26)^ The fistula origins were located in the small bowel in 3 patients, the colon in 2 patients, and unknown in 2 patients. Skin grafting was performed on average 172 days (range: 15–307) after fistula onset, once the wound condition had stabilized, using meshed grafts combined with NPWT. The graft sizes ranged from 50 to 500 cm^2^ (mean: 310 cm^2^). Graft take, which was assessed between 1 and 2 weeks postoperatively, ranged from 50% to 99%, and by 1 month, all patients had achieved complete epithelialization. Compared with these reports, our case involved a peristomal ulcer caused by mucocutaneous separation that developed on the 26th POD. The wound subsequently continued to worsen, exhibiting necrosis and instability, for about 9 weeks, and only conservative treatment could be applied during this period. Although skin grafting was eventually required due to the extensive peristomal ulcer, early intervention was not feasible because the wound bed had not yet stabilized. Once the wound stabilized, skin grafting was performed on the 90th POD. Full-thickness skin grafting combined with NPWT was then performed. Full-thickness skin grafts consist of the epidermis and the entire thickness of the dermis, containing almost all skin appendages, which provide both coverage and preservation of specialized skin functions.^28)^ They undergo minimal secondary contracture and remain robust, resulting in a more uniform texture and less likelihood of graft trauma.^28)^ In the present case, these characteristics were considered advantageous for peristomal reconstruction, where the grafted skin is exposed to repeated mechanical stress from frequent stoma appliance changes. Although both previous reports demonstrated successful wound healing without severe complications, no cases of mucocutaneous separation treated with skin grafting and NPWT have been reported, nor were there long-term follow-up observations like in our case. In our case, the patient tolerated frequent flange replacement over 6 years, and no skin problems were observed. Only a few reports on this topic have been published, and we hope that more will accumulate in the future.

This study had some limitations. First, it involved a rare case in which NPWT was combined with full-thickness skin graft transplantation to treat an ulcerated wound around a colostomy. Although mucocutaneous separation is common and may often be cured with conservative therapy, large circumferential peristomal ulceration, as seen in our case, may be rare. However, we suspect that there may be further cases that require skin grafting or NPWT, and this combined approach should be recognized as a valuable option for managing refractory peristomal ulcers. Second, NPWT may draw contaminants into the wound area, and separating the wound from the stoma requires a skillful, well-planned technique. Third, the negative pressure applied to the wound could potentially affect the intestinal wall of the stoma; therefore, careful pressure adjustment and monitoring are required. Fourth, the indications for this treatment remain unclear. However, this method may be considered a less invasive treatment option with the potential to avoid the invasive re-creation of a colostomy. In addition, it is compatible with long-term stoma care.

## CONCLUSIONS

We report that a combination of full-thickness skin graft transplantation and NPWT was successfully used to treat a patient with a large peristomal ulcer that progressively developed after complete circumferential mucocutaneous separation and infection, which occurred later than typical postoperative wound complications following neoadjuvant chemotherapy with mFOLFOX6 plus bevacizumab and laparoscopic abdominoperineal resection. Delayed wound healing, potentially related to bevacizumab, may have contributed to both the late onset and unusually extensive progression. This approach proved compatible with long-term stoma care.

## DECLARATIONS

### Fundings

No funding was received for this report.

### Availability of data and materials

The data that support the findings of this report are available on request from the corresponding author. The data are not publicly available due to ethical restrictions.
